# Childhood Obstructive Sleep Apnea and Systemic Blood Pressure and Kidney Function: A Systematic Review and Meta-Analysis

**DOI:** 10.1155/ijhy/1945725

**Published:** 2025-08-07

**Authors:** Sara Rodriguez-Lopez, Daniel Ofosu, Christopher Gerdung, Diana Keto-Lambert, Meghan Sebastianski, Meng Lin, Maria Castro-Codesal

**Affiliations:** ^1^Department of Pediatrics, University of Alberta, Edmonton, Alberta, Canada; ^2^Cummings School of Medicine, University of Calgary, Calgary, Alberta, Canada; ^3^Alberta Strategy for Patient-Oriented Research (SPOR) Support Unit, Data and Research Services, Alberta Health Services, Edmonton, Alberta, Canada

**Keywords:** diastolic blood pressure, kidney function, obstructive sleep apnea, systemic blood pressure, systolic blood pressure

## Abstract

**Background:** Obstructive sleep apnea (OSA) is a recognized risk factor for high blood pressure (BP) and chronic renal dysfunction in adults. However, it remains uncertain whether a similar association exists in children.

**Objectives:** This study assessed the associations between childhood OSA and systemic BP and renal outcomes. Additionally, it examined the effects of OSA treatments on BP in children.

**Methods:** A systematic literature search was conducted to identify relevant studies up to August 2024.

**Results:** Sixty-four studies, consisting of 44 observational studies and 20 OSA interventional studies, were included. Compared with healthy control groups, children with OSA had significantly higher daytime systolic BP (3.30 mmHg; 95% CI, 2.07–4.53), daytime diastolic BP (1.27 mmHg; 95% CI, 0.69–1.84), nighttime systolic BP (4.08 mmHg; 95% CI, 2.71–5.46), nighttime diastolic BP (2.12 mmHg; 95% CI, 0.96–3.27), daytime mean arterial pressure (MAP) (2.11 mmHg; 95% CI, 1.32–2.89), and nighttime MAP (3.60 mmHg; 95% CI, 1.11–6.09). Obesity was the only other contributing factor to daytime systolic BP elevation. Meta-analysis of studies on BP change after treatment (adenotonsillectomy or positive airway pressure) for OSA did not show significant changes in BP. Research on childhood OSA and renal outcomes is very limited.

**Conclusion:** Our results demonstrate the association between childhood OSA and higher risk of adverse systemic BP outcomes. OSA treatment alone, however, has not been demonstrated to improve BP outcomes yet. Children with OSA and systemic hypertension should be assessed for further need of BP treatment to reduce long-term cardiovascular morbidity and mortality.

## 1. Introduction

Obstructive sleep apnea (OSA) is characterized by recurrent events of partial or complete upper airway obstruction during sleep, resulting in disruption of normal ventilation and sleep patterns [1]. It has become increasingly common in children with rates up to 5.7% in the general pediatric population [2], and up to nearly 80% in children with obesity [3–5]. In adults, OSA is a known risk factor for high blood pressure (BP) [6–9], and several evidence syntheses are available [10–12].

An increasing number of publications in children have also suggested a link between OSA and high BP [13–19], although the evidence remains conflicting. Two previous meta-analyses [20, 21] have reported conflicting results regarding the association between childhood OSA and an increased risk of adverse BP outcomes. As a result, the impact of childhood OSA on BP remains unclear. Additionally, recent studies have emerged that were not included in the earlier meta-analyses. This underscores the need to summarize the latest evidence to clarify the association between childhood OSA and systemic BP.

Adenotonsillectomy (AT) is the initial treatment for children with OSA [22]. Positive airway pressure (PAP) therapy is often used to treat residual OSA after AT or if AT is not an option. This is especially true in children with obesity [22, 23]. Recent data suggest that these treatments can effectively improve BP outcomes in children with OSA [24–29]. However, there is limited research synthesizing the effects of AT on BP outcomes in children with OSA, and the results are inconsistent [30, 31]. Similarly, there is a lack of synthesis on the impact of PAP therapy on BP outcomes in children with OSA.

There are concerns among pediatric specialists about early kidney damage in children with OSA, which may also contribute to hypertension [32]. However, the association between OSA and renal outcomes in children is not as clear as in adults [33], as there is no systematic review summarizing the evidence on this topic.

The primary objective of this systematic review is to summarize the current evidence on the association between childhood OSA and systemic BP and renal outcomes. A secondary objective is to investigate the potential impact of OSA treatments on BP outcomes. This synthesis will help determine whether respiratory interventions to address OSA have the potential to improve cardiovascular health.

## 2. Methods

### 2.1. Study Design

This systematic review was conducted in accordance with the Preferred Reporting Items for Systematic Reviews and Meta-analysis (PRISMA) guidelines [34]. The protocol has been published elsewhere [35].

### 2.2. Search Strategy

The search strategy (eTable 1) consisted of terms related to OSA, systemic BP (e.g., systolic/diastolic BP [SBP/DBP] and mean arterial pressure [MAP] during sleep or wakefulness), obesity, and renal function. They were combined to create a comprehensive search in Ovid Medline and then translated into Ovid Embase, CINAHL via EBSCOhost, and Wiley Cochrane Library (which includes the Cochrane Database of Systematic Reviews, the Cochrane Central Register of Controlled Trials, the Database of Abstracts of Reviews of Effects, the Health Technology Assessment Database, and the NHS Economic Evaluation Database). A previously validated filter for studies in pediatric populations was updated and included in the search [36]. There were no language restrictions or time limits applied to the search strategy. An update of the search was run in August 2024.

### 2.3. Eligibility Criteria

We included studies that focused on children and adolescents aged 0–18 years with OSA and OSA syndrome (OSAS) as defined by the American Academy of Sleep Medicine [37]. We considered all types of observational and intervention studies for inclusion, such as controlled before–after studies, cross-sectional studies, longitudinal observational studies, case–control studies, retrospective cohort research, and case series. We included studies that had control subjects (e.g., healthy controls or primary snoring) as well as those without control subjects. Furthermore, we included studies that examined OSA interventions and those that did not.

Case reports, case series with fewer than seven subjects, abstracts, comments, editorials, letters, review articles, and animal studies were excluded.

### 2.4. Data Management and Synthesis

Records identified by the search strategy were imported into an EndNote library (Version X9, Clarivate Analytics) after removing duplicates and conducting double screening. Two researchers independently screened the titles, abstracts, and full text to identify eligible articles. Any disagreements were resolved by a third reviewer.

Using a predesigned standardized Microsoft Excel form (Microsoft, Redmond, Washington, USA), the following data items were extracted: first author, year and country of publication, study design, follow-up duration, sample size, average age or age range of study population, exclusion criteria, definition of OSA, methods of BP measurement, type of intervention, and control subjects, if applicable.

### 2.5. Quality Assessment and Certainty of Evidence

The quality assessment of the included studies was independently conducted by two reviewers using the Quality Assessment Tool for Quantitative Studies [38]. Any discrepancies were resolved through consensus. The Grading of Recommendations, Assessment, Development, and Evaluations (GRADE) approach [39] was used to assess the certainty of the evidence at the outcome level.

### 2.6. Meta-Analysis

We gathered both awake and sleep measures of SBP, DBP, and MAP for meta-analysis and outcomes were pooled using random-effects models. Continuous outcomes were summarized using the mean difference between children with OSA and healthy control group (no OSA), and statistical heterogeneity was quantified by using the *I*^2^ statistic. Publication bias was checked using regression-based Egger test. Meta-regressions were used to examine whether certain variables (mean age, mean body mass index [BMI], percentage of male participants, obesity, and hypertension) influenced the size of the difference in BP between children with OSA and control groups or the size of the intervention effect (change in BP postintervention). All the meta-analysis was performed using STATA 18 SE software (StataCorp LLC).

## 3. Results

### 3.1. Characteristics of Included Studies

**Figure 1:**
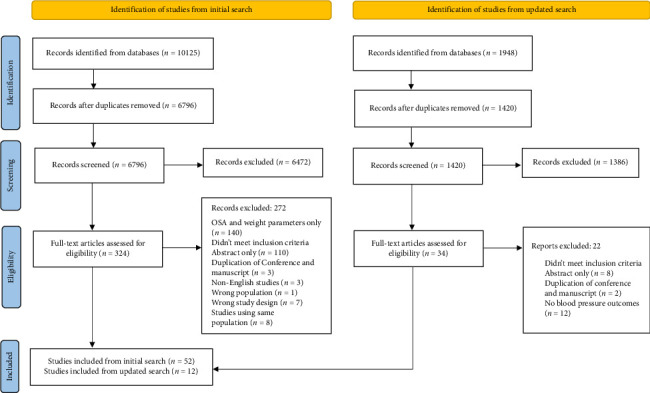
PRISMA flow diagram showing included and excluded articles.

Out of the 6796 nonduplicate records initially identified by the search strategy, 324 full texts were retrieved for eligibility assessment and 52 met the inclusion criteria. The updated search yielded 1420 articles of which 34 full texts were evaluated and 12 studies were included (Figure 1).

A total of 64 studies were included, consisting of 44 observational studies and 20 interventional studies. The majority of studies were conducted in Asia (38%) and North America (36%), with a smaller proportion from Europe (16%). Over 70% of the studies were conducted in the last decade.

The definition of OSA was based on polysomnography (PSG) parameters performed under standard recommendations [37, 40], with apnea–hypopnea index (AHI) > 1 event/hour used in most studies as per standard guidelines [41]. The majority of studies categorized OSA by severity into “mild,” “moderate,” “severe,” and “moderate/severe.” The threshold for “moderate/severe” or “severe” OSA ranged from AHI > 5 to AHI > 15. Systemic hypertension was defined by BP values > 95th percentile of standardized normative data. BP readings were reported as mean (standard deviation [SD]), median (interquartile range [IQR]), percentiles, Z-scores, or BP index.

Most studies excluded patients with a priori diagnosis of systemic hypertension or those taking medications that could affect BP. The majority of studies were classified as moderate quality (eTable 2), and the certainty of evidence for outcomes was low (eTable 3).

### 3.2. Observational Studies

Among the 44 observational studies included (Table 1), 38 were cross-sectional and six were longitudinal [43, 46, 52, 59]. Thirty-six of these studies were prospective, with sample sizes ranging from 23 to 1689 participants. Nineteen studies included healthy children as controls, while14 studies included children with primary snoring (defined as snoring and AHI < 1).

Eighteen studies measured BP using 24-h or nocturnal ambulatory BP monitoring (ABPM) [13–15, 18, 42, 45, 51, 62–67, 69, 76–78, 80], 23 measured office BP manually by sphygmomanometer or automated oscillometric device [43, 44, 46–50, 52, 53, 55–61, 68, 70–76, 81] and six studies used continuous BP measurement via finger photoplethysmography [19, 54, 57, 72, 79, 81].

### 3.3. Interventional Studies

Twenty interventional studies were included (Table 2): seven before–after prospective cohorts, six before–after retrospective cohorts, and seven randomized controlled trials (RCTs). Sixteen studies evaluated AT, three studied PAP therapy including continuous positive airway pressure (CPAP) or bilevel positive airway pressure (BPAP) [26, 89, 95], and one investigated multidisciplinary weight loss intervention [94]. Follow-up durations ranged from 3 to 24 months.

Five studies used 24-h or nocturnal BP monitoring [28, 84–89, 93], 12 measured casual BP readings [24, 26, 27, 29, 90–92, 94, 96], and three studies [82, 83, 95] did not specify the measurement method.

### 3.4. Childhood OSA and Systemic BP Outcomes

**Figure 2:**
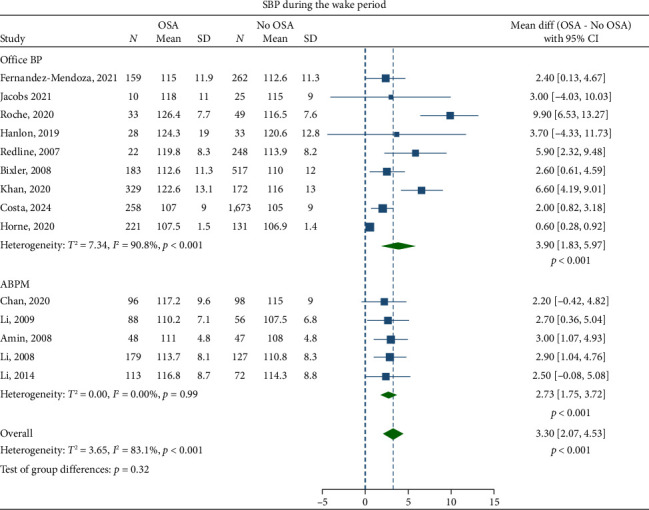
Forest plots of differences in awake systolic blood pressure (SBP) between children with obstructive sleep apnea (OSA) and healthy control group.

**Figure 3:**
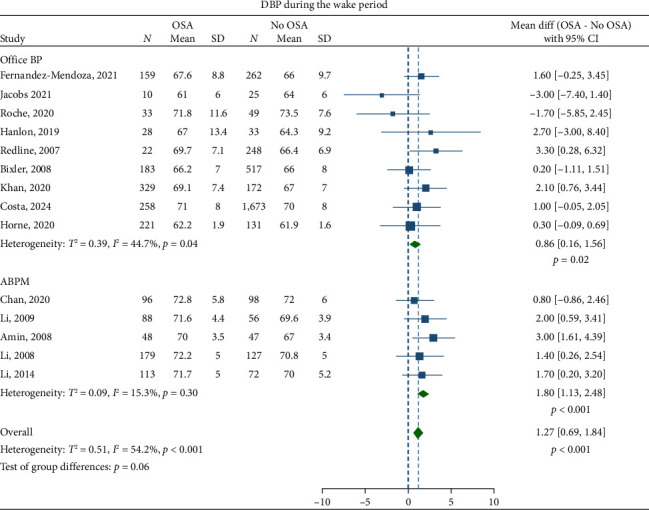
Forest plots of differences in awake diastolic blood pressure (DBP) between children with obstructive sleep apnea (OSA) and healthy control group.

**Figure 4:**
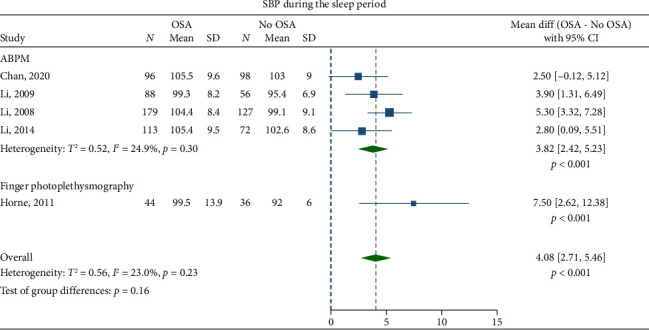
Forest plots of differences in sleep systolic blood pressure (SBP) between children with obstructive sleep apnea (OSA) and healthy control group.

**Figure 5:**
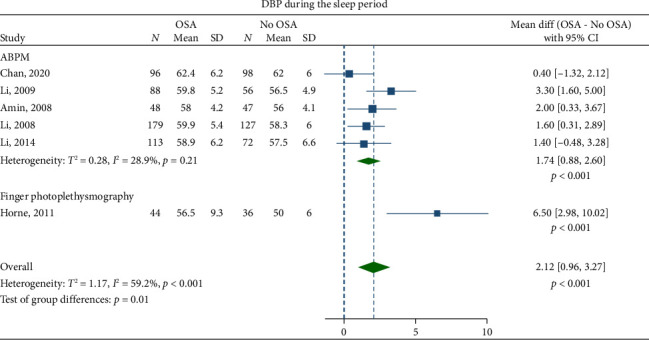
Forest plots of differences in sleep diastolic blood pressure (DBP) between children with obstructive sleep apnea (OSA) and healthy control group.

**Figure 6:**
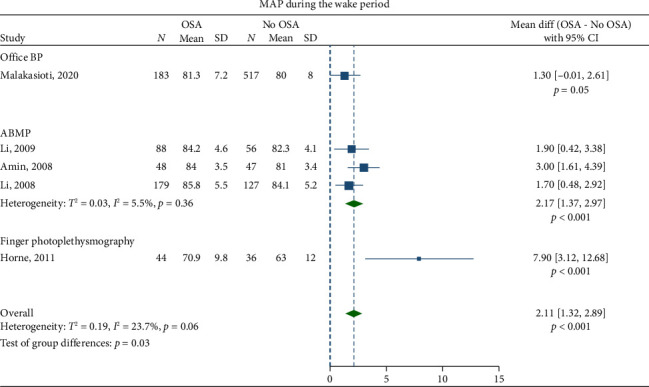
Forest plots of differences in awake mean arterial pressure (MAP) between children with obstructive sleep apnea (OSA) and healthy control group.

**Figure 7:**
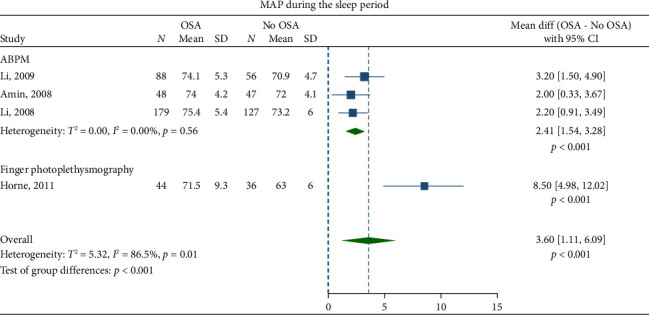
Forest plots of differences in sleep mean arterial pressure (MAP) between children with obstructive sleep apnea (OSA) and healthy control group.

Children with OSA had significantly higher awake SBP (mean difference: 3.30 mmHg; 95% CI, 2.07–4.53) and DBP (1.27 mmHg; 95% CI, 0.69–1.84) compared to the healthy control group (Figures 2 and 3). Nighttime SBP (4.08 mmHg; 95% CI, 2.71–5.46) and DBP (2.12 mmHg; 95% CI, 0.96–3.27) were also elevated in children with OSA (Figures 4 and 5). Awake MAP (2.11 mmHg; 95% CI, 1.32–2.89) and sleep MAP (3.60 mmHg; 95% CI, 1.11–6.09) were higher in children with OSA (Figures 6 and 7). ABPM provided significant evidence for BP comparisons (Figures 2, 3, 4, 5, 6, and 7).

Meta-regression analyses showed that obesity was significantly associated with an increase in awake SBP (*p*=0.003) in children with OSA. Studies with obesity rates ≤ 50% reported mean difference in awake SBP of 2.3 mmHg (95% CI, 1.4–3.3), whereas those with obesity rates > 50% reported a mean difference of 6.0 mmHg (95% CI, 3.8–8.2).

### 3.5. Effect of AT Treatment on BP Outcomes

Seven of 11 studies assessing AT on BP outcomes [24, 28, 29, 86, 88, 90, 91] reported significant BP improvements postsurgery (3–24 months). Subgroup analyses in some studies indicated greater benefits in children with baseline systemic hypertension [24, 28, 86, 88, 91] and in nonobese school-age children [90]. Three studies [28, 88, 97] showed persistence or new-onset hypertension despite improved AHI post-AT (3–6 months).

Meta-analysis of studies reporting mean BP change after AT [24, 29, 83] showed no significant differences in SBP (−0.48 mmHg; 95% CI, −11.6 to 10.2) and DBP (−2.25 mmHg; 95% CI, −12.25 to 7.74) (eFigures 1-2).

### 3.6. Effect of PAP Therapy on BP Outcomes

Three studies examined BP changes following PAP therapy. Two studies [95, 98] reported significant reductions in SBP after PAP treatment. One retrospective study [95] found adequate CPAP adherence in 33 hypertensive children with OSA was associated with a significant decrease in SBP z-score percentile (−3.5 ± 2.1 SBP percentile points per 100 days), compared to the 20 children nonadherent to CPAP, who had a 5.0 ± 1.3 increase in percentile points per 100 days. Another retrospective study [98] observed a 5-mmHg SBP reduction after six months of CPAP in a group of 25 school-age children with OSA and with or without obesity at the 6-month follow-up office visit after CPAP initiation. A subgroup analyses also found that children with a higher BMI had greater decrease in SBP. A third prospective study [89], on a cohort of 25 school-age children with moderate–severe OSA (AHI > 5 events/h) and obesity initiated on CPAP or BPAP, found no significant changes in ABPM parameters (absolute BP or nocturnal BP dipping) after 12 months of PAP therapy despite normalization of AHI.

### 3.7. Effect of Lifestyle Intervention on BP Outcomes

Roche et al. [94] evaluated a multidisciplinary weight loss intervention in 50 adolescents with obesity and with or without sleep-disordered breathing (SDB). BP improvements were observed only in participants with SDB whose condition was normalized following a 9- to 12-month weight loss intervention.

### 3.8. Childhood OSA and Renal Outcomes

Two studies [3, 99] assessed urinary albumin loss in children with OSA. Neither studies found an association between childhood OSA and albuminuria.

## 4. Discussion

This systematic review synthesized the existing literature on the association between childhood OSA and systemic BP, and kidney function. It also examined the effects of OSA treatment on BP outcomes in children. To our knowledge, this study is the most recent and largest in sample size on this topic. Additionally, it is the first to systematically analyze the impact of various OSA treatments, including PAP therapy and lifestyle modification, on BP outcomes in the pediatric population, aside from AT.

Our findings indicated that children with OSA are at a higher risk of higher BP compared to healthy non-OSA controls. However, treatment of OSA either with AT or PAP therapy alone did not always demonstrate better control of BP values on meta-analysis, despite that some studies showed encouraging results. Interventional studies continue to be limited, particularly those assessing the effect of PAP therapy and no studies were found assessing a combination of PAP and other nonsurgical treatments such as lifestyle modifications or medication management. Research on childhood OSA and renal outcomes is even more limited.

The elevated awake and sleep SBP observed in children with OSA align with prior meta-analysis that has established that moderate-to-severe childhood OSA is associated with a higher risk of adverse SBP outcomes [20]. More notably, the increased nighttime SBP and DBP further support the hypothesis that sleep disturbances associated with OSA negatively impact nocturnal BP regulation [100], a critical finding given the importance of nighttime BP as a predictor of long-term cardiovascular outcomes [101]. An important finding of this study is that the higher the prevalence of obesity in study cohorts, the greater the difference in awake SBP between children with OSA and healthy controls. This indicates that obesity exacerbates the impact of OSA on daytime BP, which is consistent with the known interaction between obesity and cardiovascular risk [61, 80, 102] in the pediatric population. The interaction and causal relationship among all three conditions is complex and likely synergistic [103, 104]. Obesity likely contributes to these differences through various mechanisms, including increased sympathetic nervous system activity, altered chemoreceptor sensitivity, and the proinflammatory state associated with excess adipose tissue [105, 106]. Children with both OSA and obesity may thus experience an amplified cardiovascular burden compared to children with OSA alone [15, 42, 55], highlighting the importance of addressing weight management in conjunction with OSA treatment to mitigate these risks.

ABPM provided consistent evidence for the BP comparisons. This is not surprising as ABPM is superior to office BP when evaluating abnormalities in BP during sleep [103], and, therefore, has shown stronger correlation with OSA [17]. ABPM identifies patients with masked hypertension and decreased physiological nocturnal dipping, an early sign of impaired BP control in children with OSA [14, 77, 80]. Performing an ABPM is, therefore, the recommended option in these children.

Evidence regarding reversible changes in BP after OSA treatment alone in children is more limited and less clear, and consistent with previous meta-analysis by Kang et al. [30] that also did not find a significant decrease in office or ABPM SBP or DBP after AT. This is not surprising considering the limited number of studies that could be meta-analyzed and the fact that most studies excluded children with previously diagnosed systemic hypertension, making it difficult to demonstrate significant impact of OSA treatment on BP outcomes. Further interventional studies in children with diagnosed systemic hypertension and OSA are, indeed, needed, to analyze the impact of OSA treatment on BP outcomes alone and/or in combination with other measures such as lifestyle changes and/or BP medication.

Another area of uncertainty is the progression of cardiovascular disease in children with OSA and BP post-OSA treatment. Some studies [28, 83, 86, 88] unexpectedly showed short-term significant increases in BP postoperatively in nonhypertensive children at baseline. An increase in BMI and somatic growth after surgery is a concern. However, no longitudinal studies have looked at the impact of OSA treatment long term as well as on the further development of hypertension during childhood and into adulthood. Further longitudinal studies are needed using appropriate measures to assess changes in BP after OSA treatment with AT, PAP therapy, or in combination with lifestyle changes and/or BP medication control.

In adults, a previous meta-analysis found that CPAP treatment was associated with a significant decrease in both day and night BP in patients with resistant hypertension [107], although with a low effect size [108]. This effect was not observed in our pediatric meta-analysis, partially due to the limited evidence available as observed in previous review [109]. Of the three studies analyzed, two reported significant improvements in SBP following PAP treatment. However, Katz's [89] study did not demonstrate a significant change in BP after PAP therapy, though there were trends indicating clinically relevant improvements in systolic BP load. The authors acknowledged that the small sample size, high attrition rate, and lack of ethnic diversity could have contributed to the lack of significant findings. Given the relatively few studies assessing the impact of PAP therapy on BP outcomes, further research is needed. Specifically, multi-ethnic cohort longitudinal studies with larger sample sizes and extended follow-ups would be beneficial to better understand the association between PAP therapy and BP outcomes. Furthermore, only 2 studies were found assessing the association between OSA and kidney outcomes, which might be due to the fact that kidney dysfunction associated with childhood OSA continues to be uncommon in children compared to adults.

Detecting and treating hypertension in children and youth is crucial to tackle its long-term cardiovascular morbidity. Previous data on BP tracking from childhood to adulthood demonstrate that higher BP in childhood correlates with hypertension in adulthood [110], a major contributor to cardiovascular morbidity and mortality worldwide [111]. Children with OSA, especially if there is concomitant obesity, should have their BP monitored, ideally by ABPM [112] for early detection of hypertension and appropriate multidisciplinary management. Conversely, children with hypertension should be assessed for symptoms of OSA, especially if there is concomitant obesity.

This systematic review was limited by the presence of moderate risk of bias in most included studies. Additionally, the included studies excluded children with prior diagnosis of hypertension or those taking antihypertensive medications, which introduces a risk of bias by potentially eliminating patients with more severe forms of hypertension. This likely reduced the likelihood of detecting significant associations. Most studies included in the analysis were observational, which may lower the certainty of the evidence presented. However, given the lack of RCTs in this field, observational studies provide valuable insights into the studied outcomes.

## 5. Conclusion

Childhood OSA is associated with deleterious BP outcomes, with obesity being a major contributor to both significant elevation of daytime SPB and OSA. OSA treatment (AT or PAP) alone, however, did not show clear effects on reducing BP outcomes, despite an encouraging trend to lower BP measures in some of the studies included in this systematic review. Given the worldwide increasing incidence of systemic hypertension in children and youth and the demonstrated correlation between childhood and adulthood hypertension, children and youth with systemic hypertension should be assessed for OSA symptoms and vice versa, especially if there is concomitant obesity, and contributing factors should be properly managed multidisciplinary.

## Figures and Tables

**Table 1:** Characteristics of observational studies included in the systematic review.

Reference (year) country	Study design	Study population	Number of participants *n* (case/control)	OSA/SDB criteria (events/hour)	Control group	Method of BP measurement
Amin et al. [14] (2004) USA	Cross-sectional, P	Children (5–17 years) referred to a pediatric sleep disorder clinic for evaluation of obstructive breathing during sleep	60 (39/21)	AHI ≥ 1	Children with primary snoring AHI < 1	ABPM
Amin et al. [42] (2008) USA	Cross-sectional, P	Children (7–13 years) from respirology clinics with snoring and hypertrophy of the tonsils and adenoids with OSA	125 (75/50)	AHI ≥ 1	Age- and gender-matched healthy children	ABPM
Archbold et al. [43] (2012) USA	Longitudinal, P	Elementary school children (6–16 years) who underwent in-home PSG initially and then 5 years later.	334	RDI ≥ 1	None	Office BP
Bixler et al. [44] (2008) USA	Cross-sectional, P	Elementary school children (5–12 years) to study sleep patterns	700 (183/517)	AHI ≥ 1	Children without OSA	Office BP
Brooks et al. [45] (2020) USA	Cross-sectional, P	Children (2–18 years) with SDB symptoms; no adenotonsillectomy	96 (78/18)	AHI ≥ 5	Children without symptoms of any SDB	ABPM
Chan et al. [46] (2020) China	Longitudinal, P	Children (6–25 years) who enrolled in an OSA study and aged 6–13 years were invited for a 10-year follow up.	243 (145/98)	OAHI ≥ 5	Children without symptoms of any SDB	ABPM
Chen et al. [47] (2024) China	Cross-sectional, R	Children (< 18 years) who underwent polysomnography for primary snoring or OSA	213	OAHI < 1	Children with primary snoring OAHI < 1	NR
Chuang et al. [48] (2021) Taiwan	Cross-sectional, R	Pediatric patients (2–17 years) with OSAS who were referred because of chronic loud snoring	396	AHI ≥ 1	None	Office BP
Chuang et al. [49] (2020) Taiwan	Cross-sectional, R	Children (2–18 years) with OSA symptoms	253	NR	None	Office BP
Costa et al. [50] (2024) Portugal	Longitudinal, P	Children (7 years) from a population-based birth cohort.	1931 (1673/238)	NR	Children without SRBD	Office BP
DelRosso et al. [51] (2021) USA	Cross-sectional, P	Children (7–18 years) referred for evaluation for sleep-disordered SDB.	41 (21/20)	OAHI ≥ 2	Age-matched children without OSA	ABPM
Fernandez-Mendoza et al. [52] (2021) USA	Longitudinal, P	Adolescents (12–23 years) who had enrolled in the Penn state child cohort as children (a random sample of elementary school children	421 (159/262)	AHI > 2	AHI < 2	Office BP
Fraire et al. [53] (2021) Argentina	Cross-sectional, P	Adolescents born in 2005 who completed the pediatric sleep Questionnaire (PSQ) at home	826	NR	None	Office BP
Geng et al. [54] (2019) China	Cross-sectional, P	Children (3–11 years) from a sleep center, referred for evaluation of habitual snoring and underwent overnight PSG	140 (43/97)	OAHI ≥ 1	Children with primary snoring OAHI < 1	Finger photoplethysmography
Hanlon et al. [55] (2019) USA	Cross-sectional, P	Children with obesity with a history of elevated BP who were referred for evaluation of hypertension.	61 (28/33)	AHI ≥ 1.5	Children without OSA	Office BP
Hinkle et al. [56] (2018) USA	Cross-sectional, R	Children from a hypertension clinic.	74 (57/17)	AHI ≥ 2	Children with primary snoring AHI < 2	Office BP
Horne et al. [19] (2011) Australia	Cross-sectional, P	Elementary school children (7–13 years) referred to a respirology clinic for suspected OSA.	80 (44/36)	AHI ≥ 1	Children without OSA and children with primary snoring	Finger photoplethysmography
Horne et al. [57] (2018) Australia	Cross-sectional, P	Children (8–18 years) attending a sleep center for assessment of suspected OSA and age-matched nonsnoring children recruited from the community	98 (74/24)	OAHI ≥ 1	Nonsnoring and children with primary snoring OAHI < 1	Office BP and finger photoplethysmography
Horne et al. [58] (2020) Australia	Cross-sectional, P	Children (3–18 years) who underwent standard pediatric overnight PSG	533 (402/131)	OAHI ≥ 1	Nonsnoring and children with primary snoring OAHI < 1	Office BP
Jacobs et al. [59] (2021) Belgium	Longitudinal, P	Children with obesity (8–18 years) recruited while entering a 12-month inpatient weight loss program	130 (87/43)	OAHI > 2	Children without OSA	Office BP
Kang et al. [18] (2015) Taiwan	Cross-sectional, P	Children (4–16 years) referred with symptoms suggestive of OSA	195 (161/34)	AHI ≥ 1	Children with primary snoring AHI < 1	ABPM
Kang et al. [60] (2022) Taiwan	Cross-sectional, P	Children (3–18 years) with OSA-related symptoms.	1689 (1039/650)	AHI > 1	Children with primary snoring (AHI < 1)	Office BP
Khan et al. [61] (2020) USA	Cross-sectional, R	Adolescents (13–21 years) referred for PSG	501 (329/172)	AHI > 1	Children without OSA	Office BP
Khan et al. [62] (2024) USA	Longitudinal, P	Children (5–14 years) with OSA and healthy control	219 (102/117)	OAHI ≥ 1	Children without OSA	ABPM
Kirk et al. [63] (2010) Canada	Cross-sectional, P	Children (4–15 years) with PSG-proven OSA recruited from a pediatric respirology clinic	30	AHI > 1.5	None	ABPM
Kohyama et al. [64] (2003) Japan	Cross-sectional, P	Children referred with suspected SDB	23	NR	None	ABPM
Kumar et al. [65] (2023) India	Cross-sectional, P	Children (5–18 years) with chronic kidney disease (stage 3–5) and nondialysis dependent	22	AHI ≥ 1	None	ABPM
Leung et al. [15] (2006) China	Cross-sectional, P	Children (6–15 years) from a sleep lab with symptoms of OSA	96	NR	None	ABPM
Li et al. [66] (2009) China	Cross-sectional, P	Non-overweight, nonobese prepubertal children (6–13 years) recruited from schools	144 (88/56)	AHI ≥ 1	Children without OSA and children with primary snoring (AHI < 1)	ABPM
Li et al. [13] (2008) China	Cross-sectional, P	Children (6–13 years) from randomly selected schools to undergo PSG	306 (179/127)	AHI > 1	Children without OSA	ABPM
Li et al. [67] (2014) China	Cross-sectional, P	Children (10–17 years) selected to undergo PSG follow-up study	127 (74/53)	AHI > 1	Children without OSA	ABPM
Malakasioti et al. [68] (2020) Greece	Cross-sectional, R	Children (3–15 years) with history of snoring and tonsillar hypertrophy without or with adenoidal hypertrophy and/or obesity	646 (491/155)	AHI > 1	Children with primary snoring (AHI < 1)	Office BP
Marcus et al. [69] (1998) USA	Cross-sectional, P	Children (> 1 year) referred to a sleep clinic for symptoms of SDB	67 (41/26)	AHI > 1	Children with primary snoring	ABPM
Martinez cuevas et al. [70] (2021) Spain	Cross-sectional, P	Children (3–14 years) with suspected sleep apnea–hypopnea syndrome (SAHS); no adenotonsillectomy or previous SAHS treatment	67 (36/31)	AHI ≥ 3	Children without SAHS	Office BP
Nisbet et al. [71] (2014) Australia	Cross-sectional, P	Children (3–5 years) referred to sleep center for symptoms of OSA	97 (62/35)	OAHI > 1	Children without OSA and children with primary snoring	Office BP and finger photoplethysmography
O'Driscoll et al. [72] (2009) Australia	Cross-sectional P	Children (7–12 years) from a sleep clinic referred for investigation of OSA	30 (20/10)	AHI ≥ 1	Children with primary snoring (AHI < 1)	Office BP and finger photoplethysmography
Reade et al. [73] (2004) USA	Cross-sectional, R	Children (4–19 years) referred for sleep study due to symptoms of OSA	90 (40/50)	AI > 1	Children without OSA	Office BP
Redline et al. [74] (2007) USA	Cross-sectional, P	Community-based cohort of adolescents (13–16 years) who underwent PSG	270 (22/248)	AHI ≥ 5	Children without OSA	Office BP
Roche et al. [75] (2020) France	Cross-sectional, P	Adolescents with obesity	82	AHI ≥ 2	None	Office BP
Tagetti et al. [76] (2017) Italy	Cross-sectional, P	Children (8–17 years) with obesity recruited from obesity clinics	39 (8/31)	OSAHS > 1.4	Children with OSAHS < 1.4	Office BP and ABPM
Weber et al. [77] (2012) Brazil	Cross-sectional, P	Children (8–12 years) from sleep disorders clinics	26 (14/12)	AHI > 4	Children with primary snoring (AHI < 4)	ABPM
Westerstahl et al. [78] (2014) Sweden	Cross-sectional, R	Children (5–13 years) with obesity followed in a specialized obesity clinic	76	AHI > 1.5	None	ABPM
Wu et al. [79] (2022) China	Cross-sectional, P	Habitually snoring children (3–14 years)	284 (199/85)	OAHI > 1	Children with primary snoring (OAHI ≤ 1)	PTT
Xu et al. [80] (2013) China	Cross-sectional, P	Children (5–14 years) with snoring referred by ENT doctors to the sleep clinic for sleep study	145 (107/38)	AHI > 5	Children with primary snoring	ABPM

Abbreviations: ABPM, ambulatory blood pressure monitoring; AHI, apnea–hypopnea index; AI, apnea index, BP, blood pressure; NR, not reported; OAHI, obstructive apnea–hypopnea index; OSA, obstructive sleep apnea; OSAHS, obstructive sleep apnea–hypopnea syndrome; P, prospective; PSG, polysomnography; PTT, pulse transit time; R, retrospective, RDI, respiratory disturbance index; SAHS, sleep apnea–hypopnea syndrome; SDB, sleep-disordered breathing; sleep; SRBD, sleep-related breathing disorders.

**Table 2:** Characteristics of interventional studies included in the systematic review.

Reference (year) country	Study design	Study population	OSA/SDB criteria (events/hour)	Type of intervention (*n*)	Control group (*n*)	Follow up months	Method of BP measurement
Apostolidou et al. [29] (2008) Greece	P, cohort. Control	Children with symptoms of OSA referred for PSG	AHI > 1	Surgery—adenotonsillectomy (58)	Children without symptoms of SDB, who were scheduled for AT for recurrent tonsillitis or recurrent otitis media (17)	5.9	Office BP
Armanac-Julian et al. [82] (2024) USA	RCT	Children (5–10 years) with OSA symptoms	AHI > 1	Surgery—adenotonsillectomy (127)	Children with OSA who are watchful waiting for surgery (128)	7	NR
Au et al. [83] (2021) China	RCT	Nonobese children (6–11 years) with mild OSA	AHI > 1	Surgery—Tonsillectomy with or without adenoidectomy and turbinate reduction. (35)	Children with OSA who are watchful waiting for surgery (36)	9	Office BP
Au et al. [84] (2023) China	RCT	Nonobese children (6–11 years) with OSA	AHI > 3	Surgery—adenotonsillectomy (62)	Children with OSA who are watchful waiting for surgery (47)	9	ABPM
DelRosso et al. [26] (2018) USA	R, cohort	Children (7–17 years) referred for symptoms of OSA	AHI ≥ 1	CPAP (25)	1. Children without OSA who snored (25) 2. Children with OSA without treatment (25)	6	Office BP
Domany et al. [27] (2021) USA	Longitudinal cohorts, P	Children (5–13 years) with OSA	OAHI > 1	Surgery—adenotonsillectomy (199)	Age- and gender-matched healthy children without OSA (174)	6–24	Office BP
Hsieh et al. [85] (2023) Taiwan	R, cohort	Children (2–17 years) with PSG-diagnosed OSAS	AHI ≥ 1	Surgery—adenotonsillectomy (245)	None	≥ 3	Office BP
Hsu et al. [86] (2018) Taiwan	P, cohort	Children (4–16 years) with symptoms suggestive of OSA	AHI ≥ 1	Surgery—adenotonsillectomy (124)	None	3.3	ABPM
Isaiah et al. [87] (2019) USA	RCT	Children (5–9 years) diagnosed with OSA	AHI > 2	Surgery—adenotonsillectomy (194)	Children with OSA who are watchful waiting for surgery (204)	7	Office BP
Kang et al. [88] (2020) Taiwan	P, cohort	Children (4–16 years) with symptoms suggestive of OSA	AHI > 1	Surgery—adenotonsillectomy-(122)	None	3–6	ABPM
Katz et al. [89] (2017) Canada	P, cohort	Children (8–16 years) with obesity and SDB	AHI ≥ 5	PAP 1. CPAP (13) 2. BPAP (14)	None	6–12	ABPM
Kuo et al. [90] (2015) Taiwan	P, case series	Children (3–18 years) with OSA-related symptoms	AHI ≥ 1	Surgery—adenotonsillectomy (78)<	None	3	Office BP
Lee et al. [24] (2018) Taiwan	R, case series	Children (< 18 years) with symptoms suggestive of OSA	AHI > 1	Surgery—adenotonsillectomy (240)	None	3–6	Office BP
Lee et al. [91] (2015) Taiwan	R, case series	Children with OSA	AHI > 1	Surgery—adenotonsillectomy (50)	None	7.5–14.5	Office BP
Ng et al. [28] (2010) China	R, cohort	Children with OSA	AHI > 1	Surgery—adenotonsillectomy (42)	None	NR	ABPM
Quante et al. [92] (2015) USA	RCT	Children (5–9.9 years) with OSA	AHI ≥ 2	Surgery within 4 weeks after randomization- adenotonsillectomy (202)	Children with OSA who are watchful waiting supportive care with reassessment for the need for surgery at 7 months (209)	7	Office BP
Redline et al. [93] (2023) USA	RCT	Children (3–12.9 years) with tonsillar hypertrophy and mild SDB	AHI < 3	Surgery—adenotonsillectomy (231)	Children with OSA who are watchful waiting for surgery (227)	12	Office BP
Roche et al. [94] (2020) France	P, cohort.	Adolescents (12–19 years) with severe obesity	AHI ≥ 2	Diet and exercise (20)	Children without SDB (30)	NR	Office BP
Shanmugam et al. [95] (2023) USA	R, cohort	Children (< 18 years) diagnosed with OSA	NR	1. CPAP (24) 2. Surgery (129). The type of surgery was not specified	None	NR	NR
Stanczyk et al. [96] (2020) Poland	P, matched cohort	Children (age median seven years) with nasopharyngeal lymphatic tissue hypertrophy, who were qualified for the surgery on a clinical basis	NR	Surgery—adenotonsillectomy (50)	Healthy children without OSA (20)	3–6	Office BP

Abbreviations: ABPM, ambulatory blood pressure monitoring; AHI, apnea–hypopnea index; AI, apnea index, BP, blood pressure; BPAP, bilevel positive airway pressure; CPAP, continuous positive airway pressure; NR, not reported; OSA, obstructive sleep apnea; OSAS, obstructive sleep apnea syndrome; P, prospective; PAP, positive airway pressure; PSG, polysomnography; R, retrospective; SDB, sleep-disordered breathing.

## Data Availability

The data that support the findings of this study are available on request from the corresponding author.

## Nomenclature

ABPM: Ambulatory blood pressure monitoring
AHI: Apnea–hypopnea index
AT: Adenotonsillectomy
BMI: Body mass index
BP: Blood pressure
BPAP: Bilevel positive airway pressure
CPAP: Continuous positive airway pressure
DBP: Diastolic blood pressure
IQR: Interquartile range
NR: Not reported
OAHI: Obstructive apnea–hypopnea index
OSA: Obstructive sleep apnea
PSG: Polysomnography
RCTs: Randomized controlled trials
RDI: Respiratory disturbance index
SBP: Systolic blood pressure
SD: Standard deviation
SDB: Sleep-disordered breathing
SAHS: Sleep apnea–hypopnea syndrome

## Supporting Information

Supporting Table 1: Search terms used for Medline and adapted for other databases.

Supporting Table 2: Quality assessment of included studies.

Supporting Table 3: GRADE evidence profile of study primary outcomes for observational studies.

Supporting Figure 1: Forest plots of the change in systolic blood pressure (SBP) after adenotonsillectomy.

Supporting Figure 2: Forest plots of the change in diastolic blood pressure (DBP) after adenotonsillectomy.
