# Supplementary material for: Childhood Obstructive Sleep Apnea and Systemic Blood Pressure and Kidney Function: A Systematic Review and Meta-Analysis
Source: Int J Hypertens. 2025 Aug 7;2025:1945725. doi: 10.1155/ijhy/1945725 (PMC12353000; doi:10.1155/ijhy/1945725)
Supplement: Supporting Information — Additional supporting information can be found online in the Supporting Information section. [file 1945725.f1.docx]

SUPPLEMENTAL TABLE 1 Search terms used for Medline and adapted for other databases

| # | Searches |
| --- | --- |
| 1 | exp Sleep Apnea Syndromes/ |
| 2 | Hypoventilation/ |
| 3 | Snoring/ |
| 4 | Sleep apn?ea*.tw,kf. |
| 5 | (sleep-disorder* adj2 breath*).tw,kf. |
| 6 | OSA.tw,kf. |
| 7 | (Hypoventilation* or hypo-ventilation*).tw,kf. |
| 8 | Pickwick*.tw,kf. |
| 9 | snoring*.tw,kf. |
| 10 | or/1-9 [MeSH & KEYWORDS FOR OSA] |
| 11 | Obesity/ |
| 12 | Obesity, Morbid/ |
| 13 | Pediatric Obesity/ |
| 14 | Body mass index/ |
| 15 | Anthropometry/ |
| 16 | Leptin/ |
| 17 | Ghrelin/ |
| 18 | (obese or obesity*).tw,kf. |
| 19 | overweight*.tw,kf. |
| 20 | body mass index.tw,kf. |
| 21 | BMI.tw,kf. |
| 22 | anthropometr*.tw,kf. |
| 23 | leptin.tw,kf. |
| 24 | ghrelin.tw,kf. |
| 25 | omentin.tw,kf. |
| 26 | or/11-25 [MeSH & KEYWORDS FOR OBESITY] |
| 27 | Hypertension/ |
| 28 | Prehypertension/ |
| 29 | Cardiovascular diseases/ |
| 30 | Blood pressure/ |
| 31 | Blood pressure monitoring, ambulatory/ |
| 32 | ((cardiac or heart*) adj2 function*).tw,kf. |
| 33 | ((cardiovascular or cardio-vascular) adj1 disease*).tw,kf. |
| 34 | (blood pressure* adj5 (high or elevated or raised or dipping or variability or nocturnal)).tw,kf. |
| 35 | Ambulatory blood pressure monitor*.tw,kf. |
| 36 | hypertensi*.tw,kf. |
| 37 | prehypertension.tw,kf. |
| 38 | nocturnal dip*.tw,kf. |
| 39 | ABPM.tw,kf. |
| 40 | SBP.tw,kf. |
| 41 | DBP.tw,kf. |
| 42 | or/27-41 [MeSH & KEYWORDS FOR HYPERTENSION] |
| 43 | exp Renal Insufficiency, Chronic/ |
| 44 | exp Proteinuria/ |
| 45 | Glomerulosclerosis, Focal Segmental/ |
| 46 | Kidney Function Tests/ |
| 47 | Glomerular Filtration Rate/ |
| 48 | Creatinine/ |
| 49 | Cystatin C/ |
| 50 | exp Renal Insufficiency/ |
| 51 | Urinalysis/ |
| 52 | Angiotensin-Converting Enzyme Inhibitors/ |
| 53 | Angiotensin II Type 2 Receptor Blockers/ |
| 54 | Proteinuria.tw,kf. |
| 55 | (Microalbuminuria or micro-albuminuria).tw,kf. |
| 56 | (protein* adj3 ratio*).tw,kf. |
| 57 | (albumin* adj3 ratio*).tw,kf. |
| 58 | Urin* PCR.tw,kf. |
| 59 | UPCR.tw,kf. |
| 60 | UACR.tw,kf. |
| 61 | Focal segmental glomerulosclerosis.tw,kf. |
| 62 | FSGS.tw,kf. |
| 63 | kidney*.tw,kf. |
| 64 | renal*.tw,kf. |
| 65 | (hyperfiltrat* or hyper-filtrat*).tw,kf. |
| 66 | glomerular filtration rate*.tw,kf. |
| 67 | (GFR or eGFR).tw,kf. |
| 68 | creatinine*.tw,kf. |
| 69 | cystatin C.tw,kf. |
| 70 | CKD.tw,kf. |
| 71 | (urine analys* or urinalys*).tw,kf. |
| 72 | (angiotensin-converting-enzyme inhibitor* or angiotensin converting enzyme inhibitor*).tw,kf. |
| 73 | ACEi.tw,kf. |
| 74 | (angiotensin adj2 receptor blocker*).tw,kf. |
| 75 | ARB.tw,kf. |
| 76 | or/43-75 [MeSH & KEYWORDS FOR KIDNEY FUNCTION] |
| 77 | Adolescent/ |
| 78 | exp Child/ |
| 79 | exp Infant/ |
| 80 | Minor/ |
| 81 | exp Pediatrics/ |
| 82 | exp Puberty/ |
| 83 | p?ediatric*.ti,ab. |
| 84 | newborn*.ti,ab. |
| 85 | neonat*.ti,ab. |
| 86 | (baby* or babies*).ti,ab. |
| 87 | (infant* or infancy).ti,ab. |
| 88 | toddler*.ti,ab. |
| 89 | (pre?school* or pre school*).ti,ab. |
| 90 | (schoolchild* or school-child*).ti,ab. |
| 91 | (schoolage* or school-age*).ti,ab. |
| 92 | nursery school*.ti,ab. |
| 93 | kindergar*.ti,ab. |
| 94 | (primary school* or grade school*).ti,ab. |
| 95 | secondary school*.ti,ab. |
| 96 | elementary school*.ti,ab. |
| 97 | (high school* or highschool*).ti,ab. |
| 98 | child*.ti,ab. |
| 99 | (kid or kids).ti,ab. |
| 100 | prepubescen*.ti,ab. |
| 101 | pubescen*.ti,ab. |
| 102 | pubert*.ti,ab. |
| 103 | adolescen*.ti,ab. |
| 104 | teenage*.ti,ab. |
| 105 | boy*.ti,ab. |
| 106 | girl*.ti,ab. |
| 107 | (minor or minors).ti,ab. |
| 108 | or/77-107 [CHILD FILTER] |
| 109 | letter/ |
| 110 | editorial/ |
| 111 | comment/ |
| 112 | case reports/ |
| 113 | review/ |
| 114 | or/109-113 [FLUFF FILTER] |
| 115 | animals/ not (humans/ and animals/) |
| 116 | (animal* or bat or bats or bovine* or calves or camel* or canine* or cat or cats or chicken* or chimp* or dog or dogs or equine* or feline* or fowl* or goat* or hamster* or horse* or llama* or mice* or monkey* or mouse* or pig or piglet* or pigs or porcine* or poultry* or primate* or rabbit* or rat or rats or rodent* or sheep* or simian* or swine* or  veterinar*).ti. |
| 117 | 115 or 116 [ANIMAL FILTER] |
| 118 | 10 and 26 and 108 |
| 119 | 10 and 42 and 108 |
| 120 | 10 and 76 and 108 |
| 121 | or/118-120 |
| 122 | 121 not 114 |
| 123 | 122 not 117 |

Supplemental Table 2 Quality assessment of included studies

| **Reference (Year)** | **Selection bias** | **Study design** | **Confounders** | **Blinding** | **Data collection method** | **Withdrawals and dropouts** | **Global rating** |
| --- | --- | --- | --- | --- | --- | --- | --- |
| Amin et al [14] (2004) | Moderate | Moderate | Moderate | Moderate | Moderate | Moderate | **Moderate** |
| Amin et al [52] (2008) | Moderate | Moderate | Moderate | Moderate | Moderate | Moderate | **Moderate** |
| Apostolidou et al [29] (2008) | Weak | Moderate | Moderate | Moderate | Moderate | Strong | **Moderate** |
| Archbold et al [45] (2012) | Weak | Moderate | Moderate | Moderate | Moderate | Moderate | **Moderate** |
| Armanac-Julian et al [94] (2024) | Moderate | Moderate | Moderate | Moderate | Moderate | Moderate | **Moderate** |
| Au et al [93] (2021) | Moderate | Moderate | Moderate | Moderate | Moderate | Moderate | **Moderate** |
| Au et al [85] (2023) | Moderate | Strong | Strong | Moderate | Moderate | Moderate | **Moderate** |
| Bixler et al [61] (2008) | Moderate | Moderate | Moderate | Moderate | Moderate | Moderate | **Moderate** |
| Brooks et al [56] (2020) | Moderate | Moderate | Moderate | Moderate | Moderate | Moderate | **Moderate** |
| Chan et al [42] (2020) | Moderate | Moderate | Moderate | Moderate | Moderate | Moderate | **Moderate** |
| Chen et al [111] (2024) | Moderate | Moderate | Moderate | Moderate | Moderate | Moderate | **Moderate** |
| Chuang et al [69] (2020) | Moderate | Moderate | Moderate | Moderate | Moderate | Moderate | **Moderate** |
| Chuang et al [70] (2021) | Moderate | Moderate | Moderate | Moderate | Moderate | Moderate | **Moderate** |
| Costa et al [76] (2024) | Moderate | Strong | Strong | Moderate | Moderate | Moderate | **Moderate** |
| DelRosso et al [26] (2018) | Moderate | Moderate | Moderate | Moderate | Moderate | Moderate | **Moderate** |
| DelRosso et al [57] (2021) | Moderate | Moderate | Moderate | Moderate | Moderate | Moderate | **Moderate** |
| Domany et al [27] (2021) | Moderate | Moderate | Moderate | Moderate | Moderate | Moderate | **Moderate** |
| Fernandez-Mendoza et al [44] (2021) | Moderate | Moderate | Moderate | Moderate | Moderate | Moderate | **Moderate** |
| Fraire et al [71] (2021) | Moderate | Moderate | Moderate | Moderate | Moderate | Moderate | **Moderate** |
| Geng et al [78] (2019) | Weak | Moderate | Moderate | Moderate | Moderate | Moderate | **Moderate** |
| Hanlon et al [64] (2019) | Weak | Moderate | Moderate | Moderate | Moderate | Moderate | **Moderate** |
| Hinkle et al [63] (2018) | Weak | Moderate | Moderate | Moderate | Moderate | Moderate | **Weak** |
| Horne et al [19] (2011) | Moderate | Moderate | Moderate | Moderate | Moderate | Moderate | **Moderate** |
| Horne et al [65] (2018) | Moderate | Moderate | Moderate | Moderate | Moderate | Moderate | **Moderate** |
| Horne et al [77] (2020) | Moderate | Moderate | Moderate | Moderate | Moderate | Moderate | **Moderate** |
| Hsieh et al [87] (2023) | Moderate | Moderate | Moderate | Moderate | Moderate | Moderate | **Moderate** |
| Hsu et al [83] (2018) | Weak | Moderate | Moderate | Moderate | Moderate | Strong | **Moderate** |
| Isaiah et al [86] (2019) | Moderate | Moderate | Moderate | Moderate | Moderate | Moderate | **Moderate** |
| Jacobs et al [43] (2021) | Moderate | Moderate | Moderate | Moderate | Moderate | Moderate | **Moderate** |
| Kang et al [18] (2015) | Moderate | Moderate | Moderate | Moderate | Moderate | Moderate | **Moderate** |
| Kang et al [72] (2022) | Moderate | Moderate | Moderate | Moderate | Moderate | Moderate | **Moderate** |
| Kang et al [84] (2020) | Weak | Moderate | Moderate | Moderate | Moderate | Strong | **Moderate** |
| Katz et al [80] (2017) | Moderate | Moderate | Moderate | Moderate | Moderate | Weak | **Moderate** |
| Khan et al [59] (2024) | Moderate | Moderate | Moderate | Moderate | Moderate | Moderate | **Moderate** |
| Khan et al [62] (2020) | Weak | Moderate | Moderate | Moderate | Moderate | Moderate | **Moderate** |
| Kirk et al [46] (2010) | Weak? | Moderate | Moderate | Moderate | Moderate | Moderate | **Moderate** |
| Kohyama et al [53] (2003) | Moderate | Moderate | Moderate | Moderate | Moderate | Moderate | **Moderate** |
| Kumar et al [58] (2023) | Moderate | Strong | Moderate | Moderate | Moderate | Moderate | **Moderate** |
| Kuo et al [89] (2015) | Weak | Moderate | Moderate | Moderate | Moderate | Strong | **Moderate** |
| Lee et al [24] (2018) | Weak | Moderate | Moderate | Moderate | Moderate | Moderate | **Moderate** |
| Lee et al [90] (2015) | Weak | Moderate | Moderate | Moderate | Moderate | Moderate | **Moderate** |
| Leung et al [15] (2006) | Moderate | Moderate | Moderate | Moderate | Moderate | Moderate | **Moderate** |
| Li et al [13] (2008) | Moderate | Moderate | Moderate | Moderate | Moderate | Moderate | **Moderate** |
| Li et al [47] (2009) | Moderate | Moderate | Strong | Moderate | Moderate | Moderate | **Moderate** |
| Li et al [54] (2014) | Moderate | Moderate | Moderate | Moderate | Moderate | Moderate | **Moderate** |
| Malakasioti et al [73] (2020) | Moderate | Moderate | Moderate | Moderate | Moderate | Moderate | **Moderate** |
| Marcus et al [55] (1998) | Moderate | Moderate | Moderate | Moderate | Moderate | Moderate | **Moderate** |
| Martinez Cuevas et al [74] (2021) | Moderate | Moderate | Moderate | Moderate | Moderate | Moderate | **Moderate** |
| Ng et al [28] (2010) | Weak | Moderate | Moderate | Moderate | Moderate | Moderate | **Moderate** |
| Nisbet et al [112] (2013) | Moderate | Moderate | Moderate | Moderate | Moderate | Moderate | **Moderate** |
| O’Driscoll et al [68] (2009) | Weak | Moderate | Weak | Moderate | Moderate | Moderate | **Weak** |
| Quante et al [91] (2015) | Moderate | Strong | Strong | Moderate | Moderate | Moderate | **Moderate** |
| Reade et al [66] (2004) | Moderate | Moderate | Moderate | Moderate | Moderate | Moderate | **Moderate** |
| Redline et al [60] (2007) | Moderate | Moderate | Moderate | Moderate | Moderate | Moderate | **Moderate** |
| Redline et al [88] (2023) | Moderate | Moderate | Moderate | Moderate | Moderate | Moderate | **Moderate** |
| Roche et al [75] (2020) | Moderate | Moderate | Moderate | Moderate | Moderate | Moderate | **Moderate** |
| Roche et al [82] (2020) | Moderate | Moderate | Moderate | Moderate | Moderate | Moderate | **Moderate** |
| Shanmugam et al [81] (2023) | Moderate | Moderate | Moderate | Moderate | Moderate | Moderate | **Moderate** |
| Stanczyk et al [92] (2020) | Moderate | Moderate | Moderate | Moderate | Moderate | Moderate | **Moderate** |
| Tagetti et al [49] (2017) | Moderate | Moderate | Moderate | Moderate | Moderate | Moderate | **Moderate** |
| Weber et al [50] (2012) | Weak | Moderate | Weak | Moderate | Moderate | Moderate | **Weak** |
| Westerstahl et al [48] (2014) | Weak | Moderate | Moderate | Moderate | Moderate | Moderate | **Moderate** |
| Wu et al [79] (2022) | Moderate | Moderate | Moderate | Moderate | Moderate | Moderate | **Moderate** |
| Xu et al [51] (2013) | Weak | Moderate | Moderate | Moderate | Moderate | Moderate | **Moderate** |

Supplemental Table 3 GRADE evidence profile of study primary outcomes for observational studies

| Certainty assessment | | | | | | | № of patients | | Effect  MD (95% CI) mmHg | Certainty |
| --- | --- | --- | --- | --- | --- | --- | --- | --- | --- | --- |
| № of studies | Study design | Risk of bias | Inconsistency | Indirectness | Imprecision | Other considerations | Children with OSA | Healthy control |  |  |
| **Daytime systolic blood pressure** | | | | | | | | | | |
| 14 | Cross-sectional studies | not serious | not serious | not serious | not serious | none | 1767 | 3508 | 3.30 (2.07 to 4.53) | ⨁⨁◯◯ Low |
| **Daytime diastolic blood pressure** | | | | | | | | | | |
| 14 | Cross-sectional studies | not serious | not serious | not serious | not serious | none | 1767 | 3508 | 1.27 (0.69 to 1.84) | ⨁⨁◯◯ Low |
| **Nighttime Systolic blood pressure** | | | | | | | | | | |
| 5 | Cross-sectional studies | not serious | not serious | not serious | not serious | none | 520 | 389 | 4.08 (2.71 to 5.46) | ⨁⨁◯◯ Low |
| **Nighttime diastolic blood pressure** | | | | | | | | | | |
| 6 | Cross-sectional studies | not serious | not serious | not serious | not serious | none | 568 | 436 | 2.12 (0.96 to 3.27) | ⨁⨁◯◯ Low |
| **Daytime mean arterial pressure** | | | | | | | | | | |
| 5 | Cross-sectional studies | not serious | not serious | not serious | not serious | none | 542 | 783 | 2.11 (1.32 to 2.89) | ⨁⨁◯◯ Low |
| **Nighttime mean arterial pressure** | | | | | | | | | | |
| 4 | Cross-sectional studies | not serious | not serious | not serious | not serious | none | 359 | 266 | 3.60 (1.11 to 6.09) | ⨁⨁◯◯ Low |

Abbreviations: CI: confidence interval; MD: mean difference


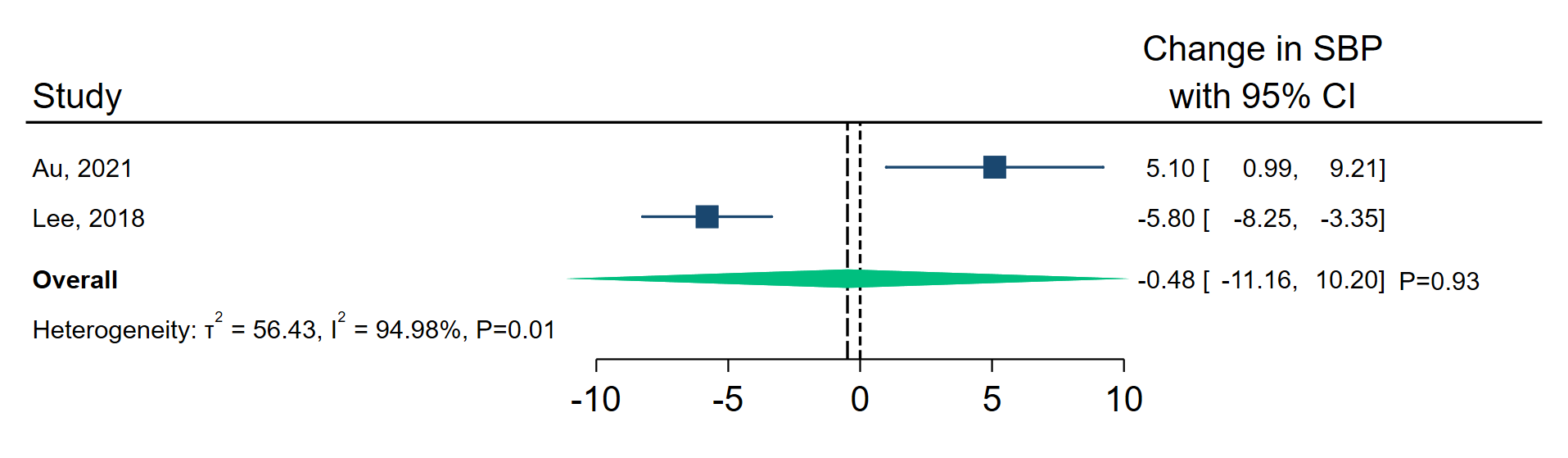


Supplemental Figure 1: Forest plots of the change in systolic blood pressure (SBP) after adenotonsillectomy


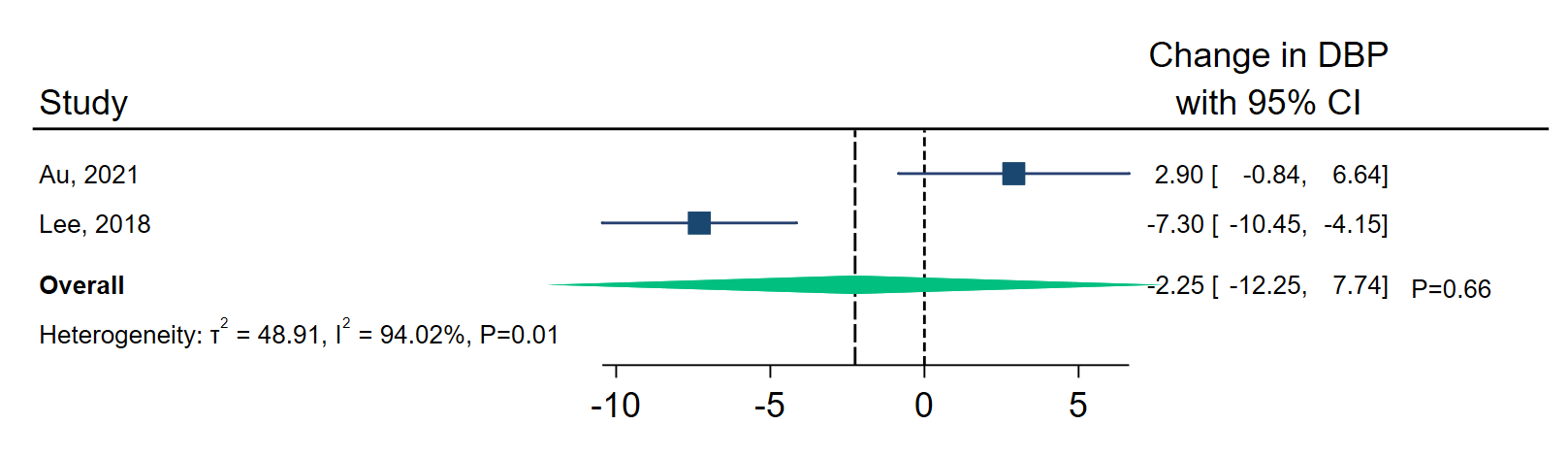


Supplemental Figure 2: Forest plots of the change in diastolic blood pressure (DBP) after adenotonsillectomy
